# Inkjet-Printed Graphene–PEDOT:PSS Decorated with Sparked ZnO Nanoparticles for Application in Acetone Detection at Room Temperature

**DOI:** 10.3390/polym16243521

**Published:** 2024-12-18

**Authors:** Ananya Thaibunnak, Suvanna Rungruang, Udomdej Pakdee

**Affiliations:** 1 Division of Printing Technology, Faculty of Science and Technology, Rajamangala University of Technology Krungthep, 2 Nanglinchi Road, Thungmahamek, Sathorn, Bangkok 10120, Thailand; ananya.t@mail.rmutk.ac.th (A.T.); suvanna.r@mail.rmutk.ac.th (S.R.); 2 Division of Physics, Faculty of Science and Technology, Rajamangala University of Technology Krungthep, 2 Nanglinchi Road, Thungmahamek, Sathorn, Bangkok 10120, Thailand

**Keywords:** inkjet printing, acetone detection, graphene, PEDOT:PSS, ZnO decoration, sparking method

## Abstract

This work presents a simple process for the development of flexible acetone gas sensors based on zinc oxide/graphene/poly(3,4-ethylenedioxythiophene)-poly(styrenesulfonate). The gas sensors were prepared by inkjet printing, which was followed by a metal sparking process involving different sparking times. The successful decoration of ZnO nanoparticles (average size ~19.0 nm) on the surface of the graphene–PEDOT:PSS hybrid ink was determined by characterizations, including Raman spectroscopy, Fourier transform infrared spectroscopy, field-emission transmission electron microscopy, X-ray photoelectron spectroscopy, and X-ray diffractometry. The ZnO nanoparticle-decorated graphene–PEDOT:PSS with a sparking time of 2 min exhibited the highest response of 71.9% at 10 ppm of acetone, above those of samples treated with other sparking times and the undecorated control. In addition, the optimal sensor revealed high selectivity for acetone over several other kinds of gases, such as ammonia, toluene, dimethylformamide, ethanol, methanol, and benzene, at room temperature. The gas sensor also revealed a low limit of detection (0.4 ppm), high sensitivity (6.18 ppm^−1^), and high stability (5-week long-term) to acetone. The response and recovery times of the sensor were found to be 4.6 min and 4.2 min, respectively. The acetone-sensing mechanism was attributed to the formation of p-n heterojunctions, which were responsible for the significantly enhanced sensitivity.

## 1. Introduction

In recent years, the volatilization of volatile organic compounds (VOCs) under atmospheric conditions has emerged as a significant issue because of the hazards to both human health and environmental integrity. Petroleum storage, perfumery, textile cleansing, and printing are among the several industrial sectors in which VOCs are involved [1,2,3,4,5]. Aromatic hydrocarbons, comprising acetone, toluene, methanol, and ethanol, are the basic VOCs that are common in human activities. Interestingly, acetone vapors have been also promoted as biomarkers that are directly correlated with physiological processes in human breath [6,7,8], which makes them particularly noteworthy. Therefore, the accurate determination of acetone concentrations is necessary for the diagnosis of an extensive variety of diseases [9,10]. Nevertheless, the technical challenge of consistently detecting acetone at low concentrations and under atmospheric conditions is significant. The traditional techniques for these types of identification are spectroscopic methods, including gas chromatography and mass spectrometry [11,12,13,14,15]. These methods tend to be restricted by their excessive operational costs. In response to these obstacles, methods of printing have recently become an attractive choice for the fabrication of gas sensors due to their cost effectiveness, high productivity, and simplicity of use [16,17,18,19,20,21]. Inkjet printing has become known as a particularly promising method for the fabrication of flexible electronic devices [22,23,24]. Inkjet ink is expelled through fine apertures to produce solution-based materials that are deposited onto substrates. The superior conductivity, chemical stability, and inherent mechanical flexibility of materials such as graphene, PEDOT:PSS, and their composites have been the subject of research for their potential applications in printed electronics [25,26]. Although many studies have documented the development of gas sensors [16,17,18,19,20,21,25], there are no reports on the application of pristine graphene–PEDOT:PSS for the detection of acetone at room temperature, to the best of our knowledge. As advanced materials for use with highly precise gas detection capacity, metal oxide semiconductors, including WO_3_ [27], In_2_O_3_ [28], Fe_2_O_3_ [29], TiO_2_ [30], and ZnO [31,32,33], have been acknowledged. The ZnO-based nanostructures have become known as a critical material for acetone detection due to their dual functionality as a transducer and receptor, which substantially improves their sensitivity to acetone [34,35,36,37]. However, the majority of ZnO-based acetone sensors require elevated operating temperatures, which typically fall within the 240–365 °C range [38,39]. The development of room-temperature acetone gas sensors that employ ZnO nanocomposites has been suggested as a viable approach to overcome these operational constraints. There are several methods available for the synthesis of ZnO nanocomposites, such as sol–gel [40], sputtering [41], evaporation [42], and hydrothermal [43] methods. In spite of the reported attractive gas-sensing properties of ZnO nanoparticles, they require a high working temperature (>200 °C) [44,45,46], which must be minimized in order to achieve practical applications with lower power consumption.

In this study, we attempted to systematically fabricate metal oxide/carbon/polymer nanocomposites composed of zinc oxide, graphene, and poly(3,4-ethylenedioxythiophene)-poly(styrenesulfonate) (ZnO@graphene–PEDOT:PSS) for acetone-sensing applications at room temperature. The nanocomposites were synthesized via a cost-efficient and new method that utilizes inkjet printing and following sparking processes. An analysis was conducted to evaluate the sensing properties of the synthesized ZnO@graphene–PEDOT:PSS nanocomposites. Furthermore, it is proposed that the acetone-sensing mechanism is optimized by the formation of p-n heterojunctions, which contribute to the increased sensitivity.

## 2. Materials and Methods

### 2.1. Preparation of Graphene–PEDOT:PSS Hybrid Ink

Graphene–PEDOT:PSS solution and several kinds of chemicals were used for the preparation of graphene–PEDOT:PSS hybrid ink. The graphene–PEDOT:PSS solution was purchased from Sigma-Aldrich Co. LLC (St. Louis, MO, USA), and comprised a 1.4% solid content and 1–3 layers of graphene sheets. The seller verified the composition of the PSS in PEDOT using a weight ratio of 5:1. Quality Reagent Chemical (QRëC^TM^) (Rawang, Malaysia) was the source of chemicals such as dimethylformamide (DMF), dimethyl sulfoxide (DMSO), ethylene glycol (EG), and Triton X-100. Volumes of 20 μL graphene–PEDOT:PSS solution and 60 μL DMF were combined to dilute the graphene–PEDOT:PSS hybrid ink. After that, the diluted solution was constantly sonicated for 2 h. Subsequently, 5.5% *v*/*v* of DMSO, 3.5% *v*/*v* of EG, and 1.0% *v*/*v* of Triton X-100 were ultrasonically sonicated to dissolve 90.0% *v*/*v* of the graphene–PEDOT:PSS solution for 30 min. Our group previously reported on the functions of DMSO, EG, and Triton X-100 in this functional ink [47,48]. Lastly, the hybrid ink was constantly blended by a stirrer with a fixed rotational speed of 1200 rpm for 2 h at room temperature.

### 2.2. Preparation of Inkjet-Printed Graphene–PEDOT:PSS Films

**Figure 1:**
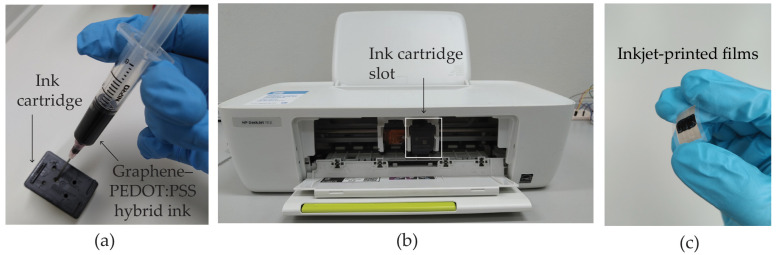
Photographs of (**a**) ink refilling and (**b**) ink cartridge slot of deskjet 1112 HP printer. (**c**) Inkjet-printed film of graphene–PEDOT:PSS on PET substrate with Ag-IDEs.

Figure 1 illustrates the sequential process of preparing inkjet-printed graphene–PEDOT:PSS films. The cartridge of an office printer (deskjet 1112 HP) was completely depleted of its original ink. Subsequently, we subjected the ink cartridge to ultrasonic cleaning with a constant frequency of 40 kHz using deionized (DI) water for 30 min, followed by drying with argon gas. Finally, we replenished it with the recently developed graphene–PEDOT:PSS hybrid ink, as shown in Figure 1a. Next, we placed the filled cartridge into the designated black-and-white (BW) cartridge slot of the printer, as shown in Figure 1b. Subsequently, the hybrid ink was applied in five successive layers onto a polyethylene terephthalate (PET) substrate together with silver interdigitated electrodes (Ag-IDEs), as shown in Figure 1c.

### 2.3. Preparation of ZnO@graphene–PEDOT:PSS Gas Sensor

**Figure 2:**
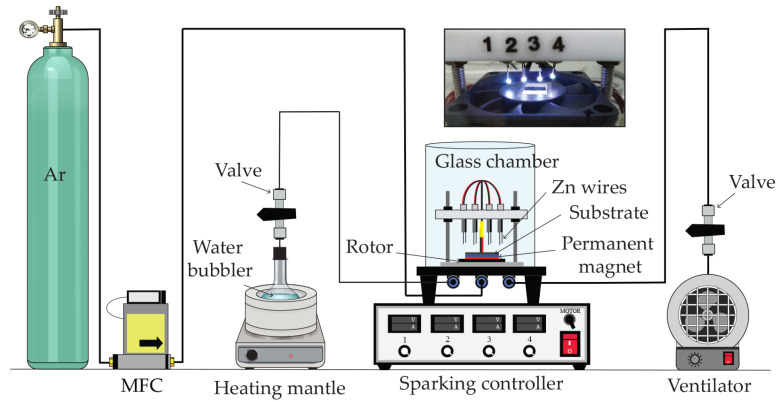
Schematic diagram of sparking system in this study.

Figure 2 shows a schematic diagram of the sparking system for producing zinc oxide (ZnO) nanoparticles on inkjet-printed graphene–PEDOT:PSS films. The sparking process involved the use of a high DC voltage to the metallic tips, leading to a melting of their surfaces. This resulted in the release and deposition of nanoparticles onto the substrate. In this study, a total of four sets of high-quality zinc wires (with a purity of 99.0% and a diameter of 0.5 mm, Advent Research Materials, Ltd., Eynsham, UK) were used as sparking tips within a glass chamber. The spacing between the zinc wires was always kept at about 2 mm above the midpoint of the substrate. The sparking system was subjected to a voltage of 3 kV and a sparking current of 10 mA. The duration of the sparking pulse and repetition rate were established at 0.2 s and 6.0 Hz, respectively. The synthesis was performed at sparking times of 1, 2, and 3 min in a stream of argon (Ar) gas, with a flow rate of 50 standard cubic centimeters per minute (sccm) controlled by a mass flow controller (MFC) (Alicat Scientific Inc., MCR-250SLPM-D/5M, Tucson, AZ, USA). In addition, water bubblers were included in the chamber, and the water flask was heated to a temperature of 65 °C using a heating mantle. In order to enhance the consistency of the distribution of the nanoparticles during the sparking process, a magnetic field from a permanent magnet was also applied and fixed at 0.3 T [49]. After the process, a ventilator was used to remove all gases to the external environment. The gas sensors developed by combining ZnO and graphene–PEDOT:PSS (ZnO@graphene–PEDOT:PSS) at sparking times of 1 min, 2 min, and 3 min were labeled as Sensor I, Sensor II, and Sensor III, respectively.

### 2.4. Characterizations and Gas-Sensing Measurements

Fourier transform infrared (FTIR) spectroscopy (Thermo Nicolet 6700, Waltham, MA, USA) and Raman spectroscopy (Horiba LabRAM HR Evolution, Kyoto, Japan) were used for analyzing the graphene–PEDOT:PSS hybrid ink. X-ray photoelectron spectroscopy (XPS) (Kratos: AXIS Supra, Manchester, UK) and field-emission transmission electron microscopy (FE-TEM: JEM 3100F, Akishima, Japan) were employed to analyze the nanostructures of the ZnO@graphene–PEDOT:PSS. An X-ray diffractometer (XRD) (Bruker D8 Discover, Karlsruhe, Germany) was also used to investigate the crystalline nature of the samples. A typical flow-through system was applied for the gas-sensing measurements; the chamber was a Swagelok with 4-way cross-fitting. Using this configuration, the ZnO@graphene–PEDOT:PSS gas sensors were evaluated for their performance in detecting a range of volatile organic compounds (VOCs) at room temperature and 54 ± 4% relative humidity, including acetone (CH_3_COCH_3_), ammonia (NH_3_), toluene (C_7_H_8_), dimethylformamide (C_3_H_7_NO), ethanol (C_2_H_6_O), methanol (CH_3_OH), and benzene (C_6_H_6_). Two flow meter controllers (Cole-Parmer, TW03227-12, Vernon Hills, IL, USA) were used to regulate the air flux at concentrations between 1 and 10 ppm. The electrical resistance of the gas sensors was measured using a 5 V voltage divider circuit that was managed by an NI USB DAQ 6008 device (National Instruments, Austin, TX, USA) at one-second intervals. LabVIEW 2018 (18.0 version) software was also used to continually record the data monitoring on a laptop. After the gas-sensing measurement, the remaining gas was extracted from the chamber by the ventilator. A schematic illustration of the gas measurement system is displayed in Figure S1.

## 3. Results and Discussion

### 3.1. Characterization of Graphene–PEDOT:PSS Hybrid Ink

**Figure 3:**
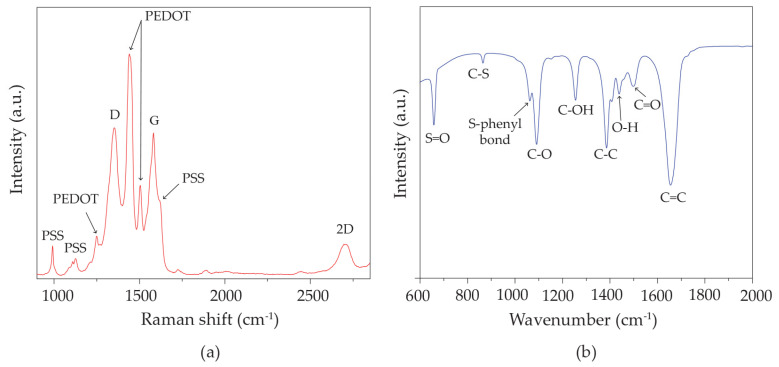
(**a**) Raman and (**b**) FTIR spectra of graphene–PEDOT:PSS hybrid ink.

Volumes of 20 μL of the graphene–PEDOT:PSS solution and 60 μL of DMF solvent were combined to dilute the graphene–PEDOT:PSS. Figure S2 shows FE-TEM images of graphene–PEDOT:PSS solutions in DMF. It was observed that some graphene sheets were overlapped and dispersed to an elevated size of ~3 nm. The overlapped graphene thickness of ~3 nm (~9 layers of graphene sheets) was confirmed by FE-TEM, as shown in Figure S2a,b. In Figure 3a, the vibrational peaks shown in the Raman spectrum of the hybrid ink consisting of graphene–PEDOT:PSS are displayed. The G-peak, exhibited at around 1580 cm^−1^, is associated with sp^2^-hybridized carbon bonds existing in the graphene structure [50]. Conversely, the D-peak, seen at around 1300 cm^−1^, is attributed to the existence of edges and defects in the graphene [50]. The peak seen at around 2620 cm^−1^ relates to a second-order two-phonon Raman vibrational mode that exhibits zone boundary defects in graphene sheets [50]. Moreover, the vibrational peaks observed for PEDOT:PSS at around 1000, 1116, 1418, 1500, and 1524 cm^−1^ may be attributed to inter-ring stretching, the distortion of C-O-C bonds, and both the asymmetrical and symmetrical stretching vibrations of the PEDOT:PSS chain [51,52].

The FTIR spectrum of the graphene–PEDOT:PSS hybrid ink, as shown in Figure 3b, reveals peaks at around 1100, 1225, 1338, and 1412 cm^−1^. These observed peaks may be referred to as the stretching modes in the C-O, C-OH, C-C, and C=O bonds, respectively [53]. Furthermore, the peaks observed at 710, 860, and 1010 cm^−1^ correspond with the S=O, C-S, and S-phenyl bonds, respectively [53]. On the other hand, the peak at 1513 cm^−1^ indicates the presence of O-H deformation vibration [53]. Therefore, it can be suggested that the graphene–PEDOT:PSS displays the successful formation of hybridized nanostructures with hydroxyl groups after the addition procedure, including DMSO, EG, and Triton X-100.

### 3.2. Characterization of Sensing Films

**Figure 4:**
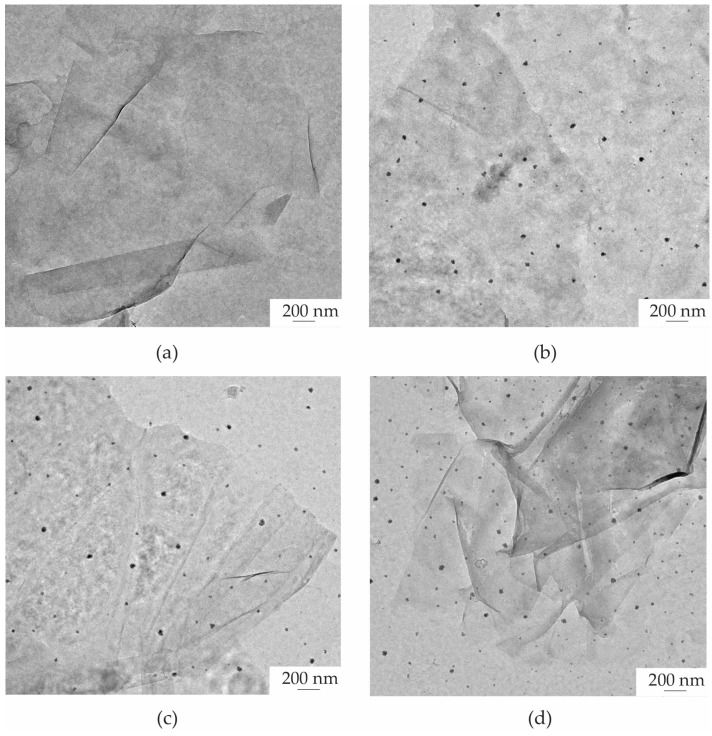
FE-TEM images of (**a**) undecorated graphene–PEDOT:PSS and ZnO-decorated graphene–PEDOT:PSS with sparking times of (**b**) 1 min, (**c**) 2 min, and (**d**) 3 min.

**Figure 5:**
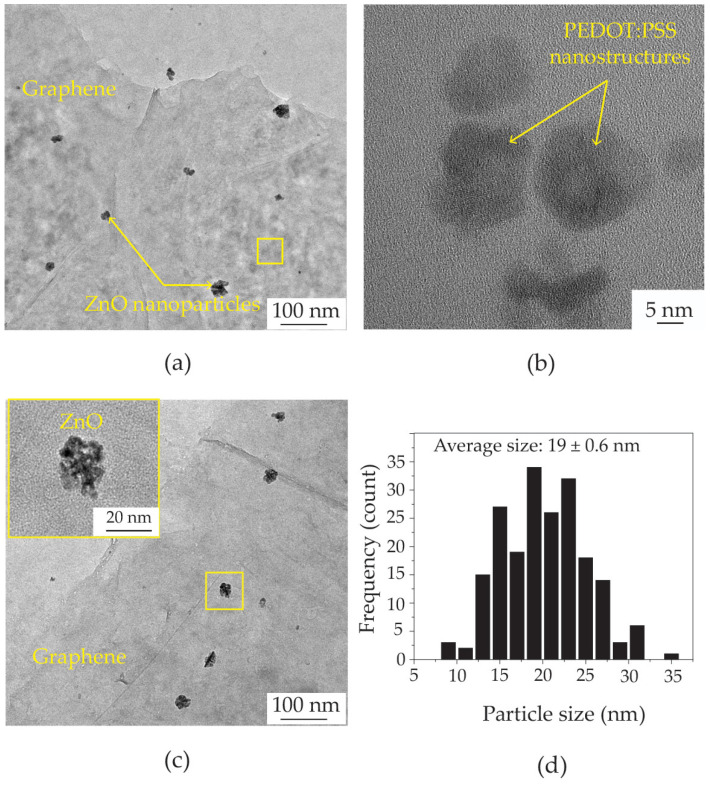
FE-TEM images of (**a**) ZnO@graphene–PEDOT:PSS nanocomposite and (**b**) PEDOT:PSS nanostructures. (**c**) FE-TEM image of ZnO@graphene–PEDOT:PSS with a high-resolution image of a ZnO nanoparticle under the condition of a 2 min sparking time. (**d**) Size distribution of ZnO nanoparticles on graphene–PEDOT:PSS films.

Figure 4 depicts the morphologies of ZnO@graphene–PEDOT:PSS for sparking times of 0, 1, 2, and 3 min. Figure 4a shows an FE-TEM image of undecorated graphene–PEDOT:PSS. After sparking procedures of 1, 2, and 3 min, the graphene–PEDOT:PSS films were decorated with nanoparticles, assumed to be ZnO, as shown in Figure 4b–d, respectively. As the sparking time increased, the ZnO nanoparticles demonstrated a slight increase in both size and density. Figure 5a,b demonstrate a systematic examination of the ZnO@graphene–PEDOT:PSS and PEDOT:PSS nanostructures with a sparking time of 2 min using FE-TEM. Figure 5c also reveals FE-TEM images of ZnO@graphene–PEDOT:PSS with a sparking time of 2 min. The inset shows a high-resolution image of a ZnO nanoparticle with a diameter size of ~19.0 nm. The histogram of the particle size distribution obtained from the FE-TEM image shows that the ZnO nanoparticles on the graphene–PEDOT:PSS nanostructures varied in size from 9 to 35 nm, with an average size of 19 ± 0.6 nm, as shown in Figure 5d.

**Figure 6:**
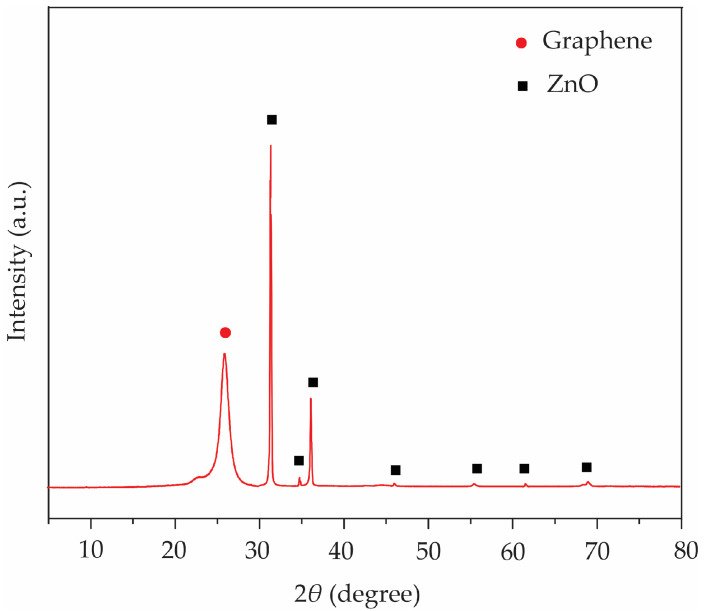
XRD pattern of ZnO@graphene–PEDOT:SS under the condition of a 2 min sparking time.

The crystalline structures of ZnO@graphene–PEDOT:SS were determined by an XRD (λ = 1.5406 Å, Cu Kα radiation). As can be seen in Figure 6, the XRD pattern of graphene peaks at 2*θ* = 26.6°, corresponding to the (002) plane, which can refer to overlapped graphene sheets or graphene flakes [54]. However, no diffraction peak could be specified for the PEDOT:PSS, indicating that it was quite amorphous [55,56]. The main diffractions of the ZnO (JCPDS No. 11-287) peaks at 2*θ* = 31.9°, 34.6°, 36.4°, 47.7°, 56.8°, 62.9°, and 68.0° were assigned to the (100), (002), (101), (102), (110), (103), and (112) crystalline planes of the ZnO nanoparticles, respectively [56,57]. The crystalline size of ZnO nanoparticles on graphene–PEDOT:PSS was calculated from Debye Scherrer’s equation, described by Equation (1) [21,55]:

(1) $$d=\frac{0.9\lambda }{\beta \cos\theta }$$

where λ is the wavelength of the X-ray source, β is the full width at half-maximum, and θ refers to the Bragg angle. The average crystalline size of the sample was calculated to be 17.8 nm. This calculated value is in close agreement with the results obtained from FE-TEM images (see Figure 5c).

**Figure 7:**
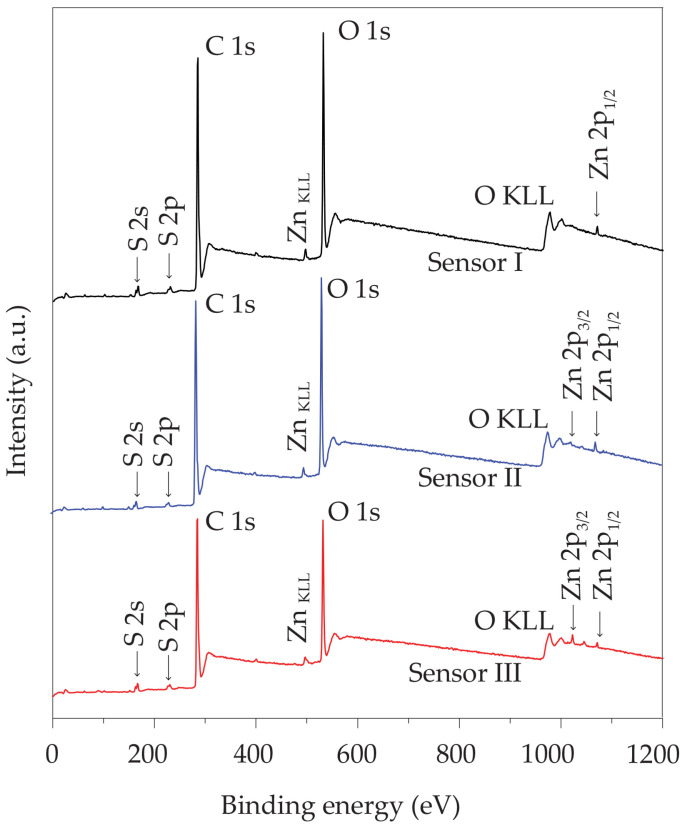
Survey-scan XPS spectra of ZnO@graphene–PEDOT:PSS gas sensors at sparking times of 1 min (Sensor I), 2 min (Sensor II), and 3 min (Sensor III).

Figure 7 displays the examination of the chemical compositions on the surfaces of ZnO@graphene–PEDOT:PSS gas sensors exposed to sparking times of 1 min, 2 min, and 3 min (referred to as Sensor I, Sensor II, and Sensor III, respectively), using XPS. The presence of carbon (C), oxygen (O), sulfur (S), and zinc (Zn) was noted in the XPS spectra obtained by a survey scan of Sensors I, Sensor II, and Sensor III. The chemical compositions of ZnO@graphene–PEDOT:PSS can be verified through these substances. The Zn contents of Sensors I, Sensor II, and Sensor III obtained from XPS measurements were found to be 1.6 wt%, 2.9 wt%, and 6.9 wt%, respectively. Therefore, the concentration of Zn increased as the duration of the sparking process increased.

**Figure 8:**
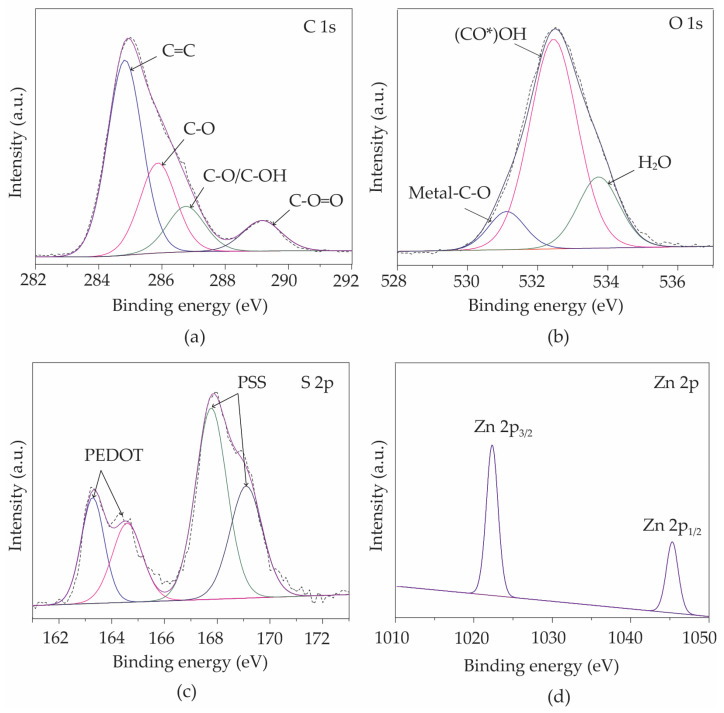
High-resolution XPS spectra of Sensor II for (**a**) C 1s, (**b**) O 1s, (**c**) S 2p, and (**d**) Zn 2p core levels.

Figure 8 presents high-resolution XPS spectra for the core levels of C 1s, O 1s, S 2p, and Zn 2p, derived from Sensor II. The chemical bonds can be identified by deconvoluting the spectra using Gaussian–Lorentzian curve fitting. As can be seen in Figure 8a, the decomposition of the C 1s peak produced four components at binding energies of 284.8 eV, 285.9 eV, 286.9 eV, and 289.1 eV, corresponding to C=C, C-O, C-O/OH, and C-O=O bonds [58,59], respectively. The C=C component is correlated with the sp^2^ hybridized structure of graphene, while the C-O, C-O/OH, and C-O=O components are most likely the result of interactions between ZnO nanoparticles and graphene nanostructures after the sparking procedure. Three prominent characteristics of the O 1s peak in Figure 8b can be observed at binding energies of 531.2 eV, 532.7 eV, and 534.2 eV. These characteristics are associated with metal–C-O, (CO*)OH, and chemisorbed H_2_O, respectively [50]. The existence of the metal–C-O bond, which is expected to be the Zn-C-O bond, suggests the effective bonding of ZnO onto the graphene–PEDOT:PSS nanostructures. A spectral analysis of the S 2p spectrum in Figure 8c revealed the existence of PEDOT:PSS in Sensor II. Binding energies from 163 eV to 166 eV are associated with sulfur (S 2p_3/2_) in PEDOT [60], whereas energies ranging from 167 eV to 170 eV correspond to sulfur in PSS (S 2p_1/2_) [60]. As shown in Figure 8d, the Zn 2p core-level spectra of Sensor II display two separate peaks at 1022.3 eV and 1045.3 eV, which correlate to the Zn^2^⁺ oxidation state in ZnO nanoparticles [61].

### 3.3. Gas-Sensing Properties

**Figure 9:**
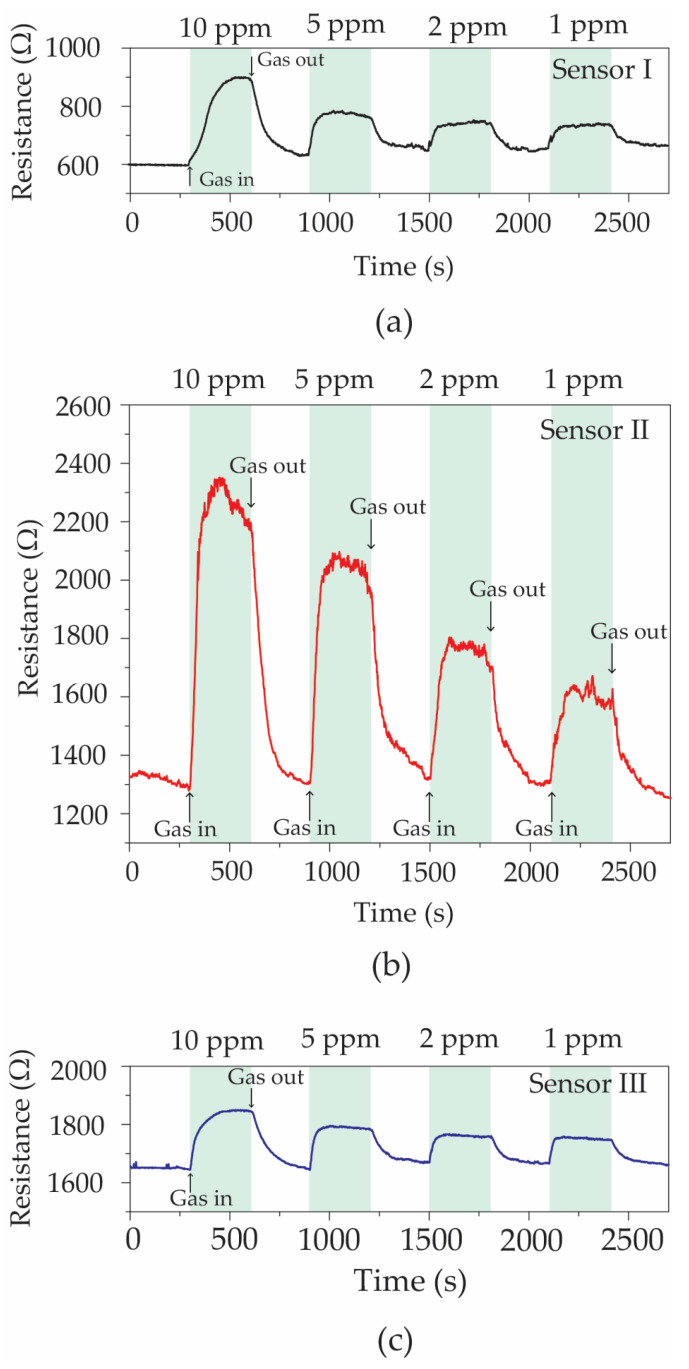
Variation in the resistances of (**a**) Sensor I, (**b**) Sensor II, and (**c**) Sensor III subjected to acetone concentrations of 1, 2, 5, and 10 ppm at room temperature.

Figure 9 displays the variations in electrical resistance of Sensor I (Figure 9a), Sensor II (Figure 9b), and Sensor III (Figure 9c) under acetone concentrations of 1, 2, 5, and 10 ppm at room temperature. Upon exposure to acetone vapor, all gas sensors demonstrated an increase in resistance and afterwards returned to their baseline levels in dry air. The results of the experiment indicate that all the ZnO@graphene–PEDOT:PSS sensors have p-type semiconducting properties when exposed to acetone vapor as a reducing gas [20,47,48,53]. In addition, Sensor II, prepared with a sparking time of 2 min, displayed a greater response to acetone in comparison to sensors with sparking times of 1 min and 3 min, regardless of the acetone concentration investigated. An analysis of the data indicated that the optimal condition for the ZnO sparking process is a time of 2 min. The gas response, sensitivity, and selectivity properties of all the sensors were evaluated to determine their performances. The gas response is quantitatively described by Equation (2) [47,48,53,54,55,56,57,58,59]:

(2) $$S\left(\%\right)=\frac{R_{\mathrm{gas}}-R_{\mathrm{air}}}{R_{\mathrm{air}}}\times 100,$$

where R_gas_ and R_air_ are the electrical resistances of the gas sensor in test gas and dry air, respectively. The gas response values calculated for the optimal ZnO@graphene–PEDOT:PSS gas sensor at acetone concentrations of 1, 2, 5, and 10 ppm were 18.8%, 27.8%, 47.8%, and 71.9%, respectively. It should be noted that the majority of gas sensors demonstrate limited sensitivity to acetone at room temperature. Therefore, the results obtained in this work are quite impressive and highlight the outstanding capability of the sensor under ambient conditions.

**Figure 10:**
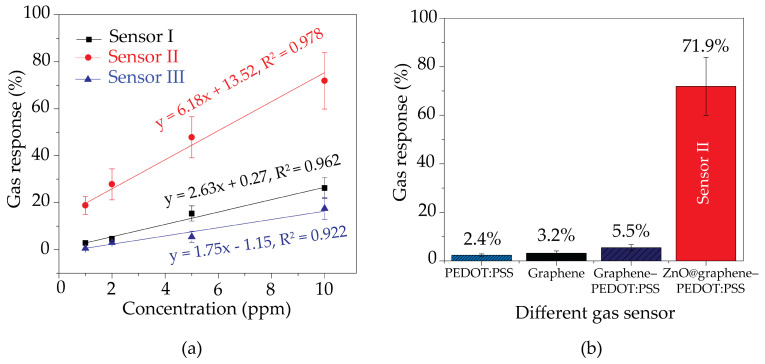
(**a**) Gas responses of all fabricated gas sensors as functions of the acetone concentration at room temperature. (**b**) Gas response to 10 ppm acetone vapor of Sensor II compared with those of PEDOT:PSS, graphene, and graphene–PEDOT:PSS at room temperature.

The sensitivity characteristic is determined by the slope of the linear graph of the relationship between the gas response and gas concentration [47,48,58,61]. The selectivity property was investigated by comparing the responses of the gas sensor to several VOC gases [47,48,57,58,61]. Figure 10a shows the gas response values for Sensor I, Sensor II, and Sensor III in relation to the concentration of acetone. For the entire range of concentrations, the responses exhibit a nearly linear relationship. The slope of the linear function indicates the rate of change in response with respect to the concentration, which is also an indicator of sensitivity. The results suggest that the sensors developed with 1 min and 3 min sparking times possess sensitivities of 2.63 ppm^−1^ and 1.75 ppm^−1^, respectively, in contrast to Sensor II, which provides the highest sensitivity at 6.18 ppm^−1^. To determine the limit of detection (LOD) for the sensors, the equation 3σ/S can be defined [57]. In this equation, the slope of the linear function is indicated by S, while the relative standard deviation is denoted by σ. The LOD values of Sensor I, Sensor II, and Sensor III were recently determined to be 1.1, 0.4, and 2.4 ppm, respectively. The response time can be defined as the time of 90% resistance change for the sensor after a gas-sensing cycle. The recovery time of the sensor can be further defined as the time of resistance change and recovery to its baseline level. The response and recovery times of the sensors for all experiments were found to be ~4.6 min and ~4.2 min, respectively. A further investigation was carried out to examine the impact of ZnO decoration on the gas response to 10 ppm acetone vapor at room temperature. It is shown in Figure 10b that the gas responses of PEDOT:PSS, graphene, graphene–PEDOT:PSS, and Sensor II were 2.4%, 3.2%, 5.5%, and 71.9%, respectively. Therefore, the gas response was substantially enhanced from 2.4% to 71.9% through the addition of graphene and ZnO nanoparticles into the PEDOT:PSS polymer. However, the addition of more ZnO into the graphene–PEDOT:PSS nanocomposites led to a decrease in the acetone response, as shown in Figure 9c. Consequently, Sensor II is the most efficient configuration of the ZnO@graphene–PEDOT:PSS gas sensor in terms of detecting acetone vapor at room temperature.

The gas response of Sensor II improves as the Zn loading content increases, and this enhancement is directly correlated with the time of Zn sparking. The observed enhancement may be related to the greater number of ZnO nanoparticles, which encourage the creation of more p-n heterojunctions on the graphene–PEDOT:PSS surface [50]. Nevertheless, if the metal loading concentration reaches its limit (Sensor III), the ZnO molecules may aggregate into bigger nanoparticles. This process of accumulation results in a reduction in the density of p-n heterojunctions, which leads to a decrease in the acetone response at room temperature.

**Figure 11:**
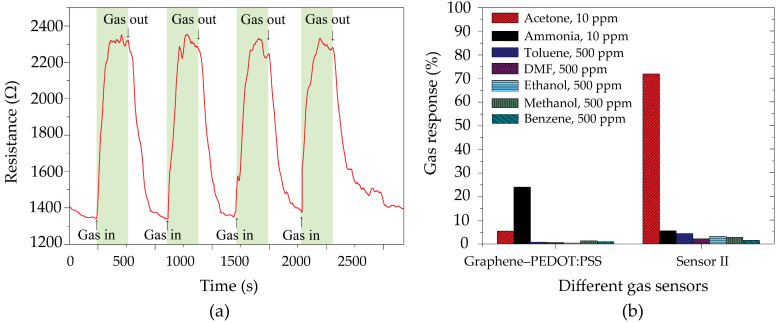
(**a**) Four repeated pulses of Sensor II toward 10 ppm acetone vapor and (**b**) selectivity histogram of graphene–PEDOT:PSS gas sensor and Sensor II to different VOCs at room temperature.

The reproducible characteristics of Sensor II were assessed by subjecting it to a 10 ppm concentration of acetone vapor at room temperature, as illustrated in Figure 11a. Sensor II demonstrated a complete return to its initial baseline after four experimental cycles. The selectivity properties of both the graphene–PEDOT:PSS gas sensor and Sensor II for a variety of analytes, including 10 ppm acetone, 10 ppm ammonia, 500 ppm toluene, 500 ppm dimethylformamide, 500 ppm ethanol, 500 ppm methanol, and 500 ppm benzene, were carefully investigated, as shown in Figure 11b. Sensor II exhibited a significant selectivity for acetone in comparison with the other gases at room temperature, while the graphene–PEDOT:PSS gas sensor displayed a notable selectivity for ammonia. The selectivity of the graphene–PEDOT:PSS gas sensor toward NH_3_ may be due to the chemical reactions between ammonia molecules and hydroxyl groups on the graphene–PEDOT:PSS surface. In addition, direct charge transfer processes between the undecorated graphene–PEDOT:PSS surface and gas molecules are also considered a sensing mechanism [47,48,53].

The acetone-sensing performance of Sensor II is superior to that in previous research, as shown in Table 1. It should be noted that most nanomaterials cannot be sensitive to acetone molecules at room temperature. A previous study demonstrated a sensor with higher acetone responsiveness, but its high operating temperature remains a significant restriction [38]. The stability property of Sensor II was assessed in a period of 8 weeks under the same working conditions. An essentially slight decrease in gas response (about 1.9% from the initial value) was observed after 5 weeks of storage at room temperature in an environment with uncontrolled relative humidity (RH). However, it is shown in Figure S3 that the gas response of the sensor declined rapidly after week 6 (60.1%) and collapsed at week 8 (22.3%).

**Figure 12:**
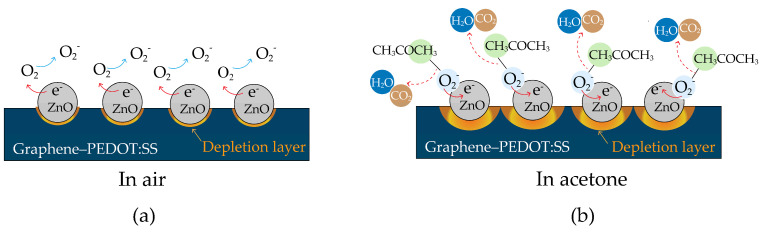
Schematics of the proposed acetone-sensing mechanism of ZnO@graphene–PEDOT:PSS gas sensor after contact with (**a**) air and (**b**) acetone at room temperature.

Figure 12 displays the acetone-sensing mechanism of Sensor II as a ZnO@graphene–PEDOT:PSS gas sensor, which can be explained by the formation of p-n heterojunctions between the p-type graphene–PEDOT:PSS and n-type ZnO nanoparticles. Upon exposure of graphene–PEDOT:PSS to air, oxygen molecules ($O_{2}$) attach to the surface and receive electrons from the valence band, resulting in the appearance of oxygen species ($O_{2}^{-}$) via the reaction $O_{2}(ads)+e^{-}\to O_{2}^{-}(ads)$. The impact of this phenomenon is a reduction in electrical resistance and an increase in the density of holes. At the interface between the n-type ZnO nanoparticles and the p-type graphene–PEDOT:PSS, p-n heterojunctions are formed after the deposition of ZnO onto the graphene–PEDOT:PSS surface. The effective transfer of electrons from ZnO to graphene–PEDOT:PSS is made possible by the differences in their work functions, leading to the formation of depletion layers. Figure 12a displays this feature by showing a systematic increase in electrical resistance throughout the graphene–PEDOT:PSS structure [34,66]. Additionally, there has been research reporting that the formation of p-n heterojunctions can also promote the enhancement of the response and recovery times of a sensor [67].

Upon exposure to acetone vapor, which behaves as a reducing gas, a chemical reaction arises between the acetone molecules and the oxygen species, causing the following reaction: $CH_{3}COCH_{3}{(\mathrm{gas})+4O}_{2}^{-}(ads)\to 3CO_{2}+3H_{2}O+4e^{-}$ [50,68]. By this procedure, electrons are released into the valence band of graphene–PEDOT:PSS. After recombination with holes in the graphene–PEDOT:PSS, these electrons promote an increase in the width of the depletion layer, depicted in Figure 12b. Moreover, the concentration of holes in graphene–PEDOT:PSS decreases when it reacts with acetone, leading to an increase in the potential barrier height (Δϕ). This behavior is described by the equation R = Aexp(Δϕq/kT), where R represents the sensor resistance, A is a constant, q is the electron charge, Δϕ is the potential barrier height, k is Boltzmann’s constant, and T is the temperature of the sensing layer [50,55,57,69]. Therefore, as the height of the potential barrier for the sensor increases, the electrical resistance also increases.

## 4. Conclusions

We successfully fabricated ZnO@graphene–PEDOT:PSS gas sensors at room temperature using inkjet printing followed by the sparking method and systematically characterized them for acetone sensing. Raman and FTIR spectroscopy confirmed the chemical composition and bonding characteristics of the hybrid ink based on graphene and PEDOT:PSS. The presence of ZnO nanoparticles with the graphene–PEDOT:PSS nanostructures was further substantiated by FE-TEM and XPS analyses. The ZnO@graphene–PEDOT:PSS gas sensor suggested p-type semiconducting behavior in response to acetone, as indicated by the gas-sensing results. Notably, the acetone sensitivity was significantly improved at room temperature as a result of the optimization for the 2 min sparking time with the addition of ZnO nanoparticles. The ZnO@graphene–PEDOT:PSS gas sensor demonstrated superior acetone sensitivity at a concentration of 10 ppm in comparison to graphene, graphene–PEDOT:PSS, and PEDOT:PSS gas sensors. In addition, these sensors revealed an important selectivity for acetone over several other kinds of VOC gases at room temperature. The p-n heterojunctions created between n-type ZnO nanoparticles and p-type graphene–PEDOT:PSS nanostructures were responsible for the acetone-sensing mechanism of the ZnO@graphene–PEDOT:PSS nanostructure. This finding may be accurately used across various industries, including petroleum storage, perfumery, textile purification, and printing, for the monitoring of acetone.

## Figures and Tables

**Table 1:** Performance comparison of acetone gas sensors.

Materials	Operating Temperature (°C)	Response (%)	Response Definition	Acetone Concentration (ppm)	Ref.
Fe-doped ZnO	365	105.70	R_a_/R_g_	100	[38]
Cd/Mn-doped ZnO	240	11.40	R_a_/R_g_	20	[39]
In_2_O_3_/MWCNTs	300	15.97	R_a_/R_g_	250	[62]
SnO_2_/Co_3_O_4_ nanotubes	140	2.70	R_g_/R_a_	10	[63]
GO/TiO_2_	RT	18.66	(ΔI/I_o_) × 100	200	[64]
CdS-doped TiO_2_	RT	71.00	(ΔR/R_air_) × 100	5000	[65]
ZnO@graphene–PEDOT:PSS (Sensor II)	RT	71.90	(ΔR/R_air_) × 100	10	This work

## Data Availability

Data are contained within the article and the Supplementary Materials.

## Supplementary Materials

The following supporting information can be downloaded at: https://www.mdpi.com/article/10.3390/polym16243521/s1, Figure S1: Schematic diagram of gas–sensing measurement setup; Figure S2: FE-TEM images of graphene-PEDOT:PSS in DMF with (a) low resolution and (b) high resolution; Figure S3: The measurements of the gas response for the optimal ZnO@graphene–PEDOT:PSS gas sensor in a period of 8 weeks upon repetitive exposure to 10 ppm acetone at room temperature.
