# Supplementary material for: Inkjet-Printed Graphene–PEDOT:PSS Decorated with Sparked ZnO Nanoparticles for Application in Acetone Detection at Room Temperature
Source: Polymers (Basel). 2024 Dec 18;16(24):3521. doi: 10.3390/polym16243521 (PMC11677939; doi:10.3390/polym16243521)
Supplement: Supplementary file 1 [file polymers-16-03521-s001.zip › polymers-3352374-supplementary.pdf]

Supplementary Materials

Inkjet-Printed Graphene-PEDOT:PSS Decorated with Sparked ZnO Nanoparticles for Application in Acetone Detection at Room Temperature

Ananya Thaibunnak [1], Suvanna Rungruang [1] and Udomdej Pakdee [2,*]

[1] Division of Printing Technology, Faculty of Science and Technology, Rajamangala University of Technology Krungthep, 2 Nanglinchi Road, Thungmahamek, Sathorn, Bangkok, 10120, Thailand; ananya.t@mail.rmutk.ac.th (A.T.); suvanna.r@mail.rmutk.ac.th (S.R.)

[2] Division of Physics, Faculty of Science and Technology, Rajamangala University of Technology Krungthep, 2 Nanglinchi Road, Thungmahamek, Sathorn, Bangkok, 10120, Thailand;

*Correspondence: udomdej.p@mail.rmutk.ac.th (U.P.)

Contents:

1. Schematic diagram of gas-sensing measurement setup.

2. Field emission transmission electron microscope images of graphene-PEDOT:PSS in DMF.

3. The measurements of the gas response for the optimal ZnO@graphene-PEDOT:PSS gas sensor in a period of 8 weeks upon repetitive exposure to 10 ppm acetone at room temperature.

1. Schematic diagram of gas-sensing measurement setup.

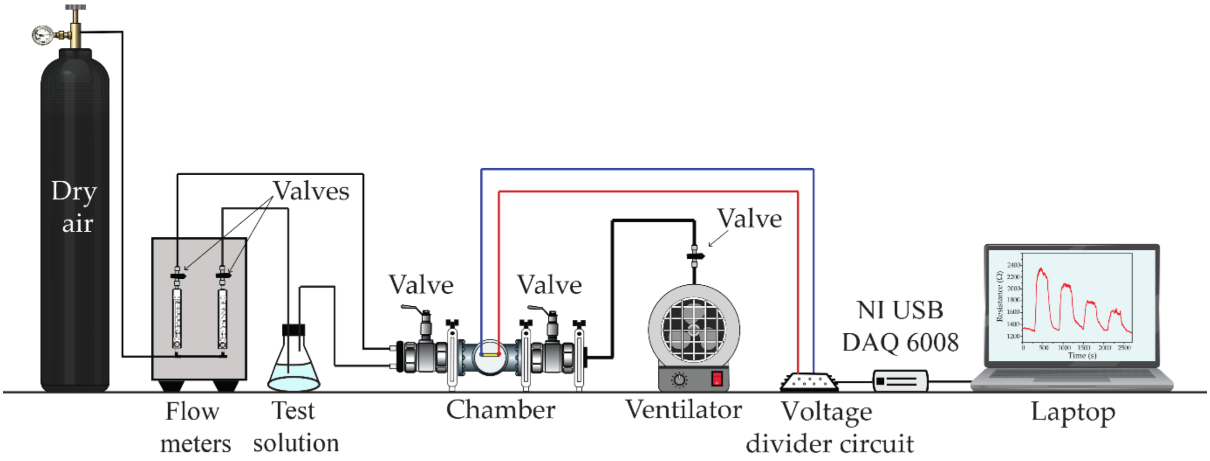

Figure S1. Schematic diagram of gas-sensing measurement setup.

2. Field emission transmission electron microscope images of graphene-PEDOT:PSS in DMF.

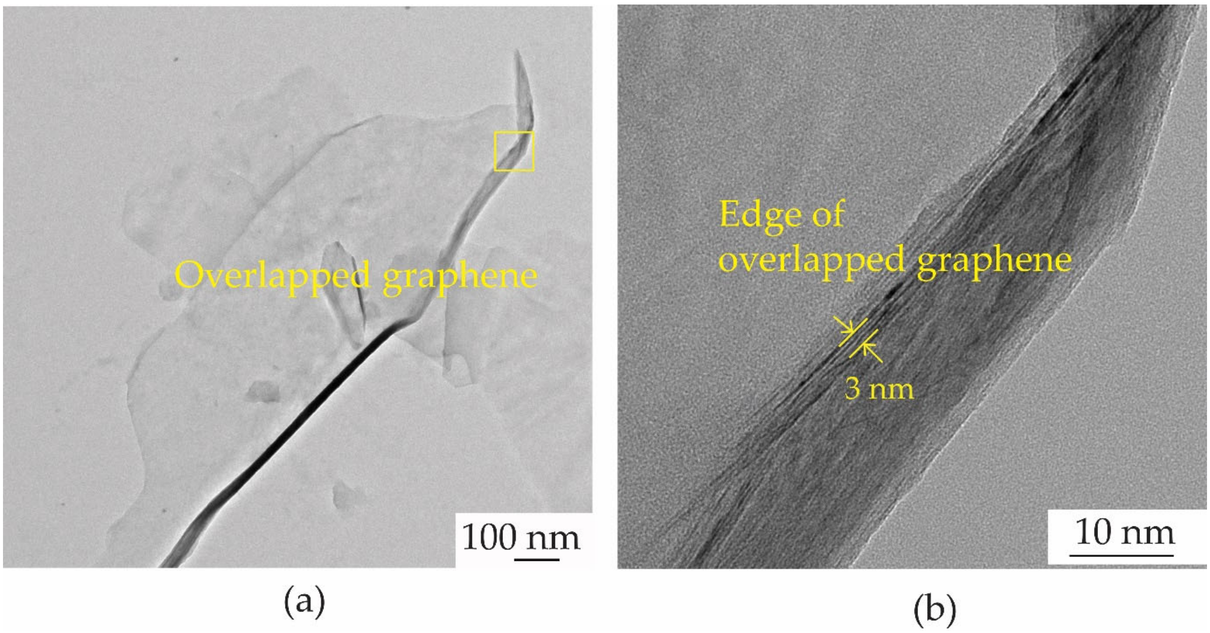

Figure S2. FE-TEM images of graphene-PEDOT:PSS in DMF with (a) low resolution and (b) high resolution.

3. The measurements of the gas response for the optimal ZnO@graphene-PEDOT:PSS gas sensor in a period of 8 weeks upon repetitive exposure to 10 ppm acetone at room temperature.

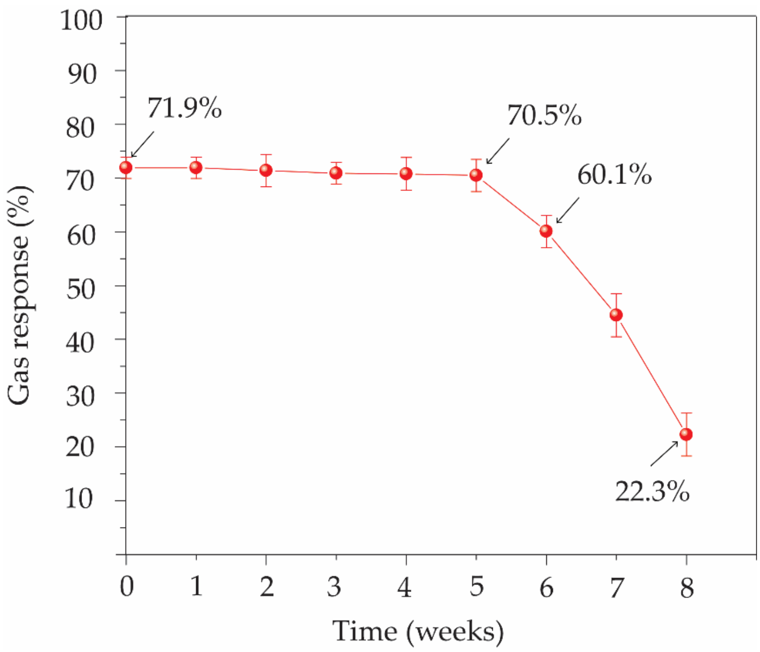

Figure S3. The measurements of the gas response for the optimal ZnO@graphene-PEDOT:PSS gas sensor in a period of 8 weeks upon repetitive exposure to 10 ppm acetone at room temperature.
